# Monitoring Snake Venom-Induced Extracellular Matrix Degradation and Identifying Proteolytically Active Venom Toxins Using Fluorescently Labeled Substrates

**DOI:** 10.3390/biology12060765

**Published:** 2023-05-24

**Authors:** Mátyás A. Bittenbinder, Nick D. Bergkamp, Julien Slagboom, Jan Paul M. Bebelman, Nicholas R. Casewell, Marco H. Siderius, Martine J. Smit, Jeroen Kool, Freek J. Vonk

**Affiliations:** 1 Naturalis Biodiversity Center, 2333 CR Leiden, The Netherlandsf.j.vonk@vu.nl (F.J.V.); 2 Division of BioAnalytical Chemistry, Department of Chemistry and Pharmaceutical Sciences, Amsterdam Institute of Molecular and Life Sciences (AIMMS), Faculty of Sciences, Vrije Universiteit Amsterdam, 1081 HZ Amsterdam, The Netherlands; 3 Centre for Analytical Sciences Amsterdam (CASA), 1081 HZ Amsterdam, The Netherlands; 4 Division of Medicinal Chemistry, Amsterdam Institute of Molecular and Life Sciences (AIMMS), Vrije Universiteit Amsterdam, 1081 HZ Amsterdam, The Netherlands; 5 Centre for Snakebite Research & Interventions, Liverpool School of Tropical Medicine, Pembroke Place, Liverpool L3 5QA, UK

**Keywords:** snakebite, tissue-damaging activities, extracellular matrix, protease, SVMP

## Abstract

**Simple Summary:**

Snakebite envenoming is an important public health issue with annual mortality rates ranging between 81,000 and 138,000. Snake venoms may cause a range of pathophysiological effects and may have tissue-damaging activities that result in lifelong morbidities. The tissue-damaging components in snake venoms comprise multiple toxin classes with various molecular targets including cellular membranes and the extracellular matrix (ECM). In this study, we present multiple assay formats that enable us to study ECM degradation using a variety of fluorescently labeled ECM components. This workflow could provide valuable insights into the key mechanisms by which proteolytic venom components exert their effects. The workflow could prove useful for the development of effective snakebite treatments.

**Abstract:**

Snakebite envenoming is an important public health issue with devastating consequences and annual mortality rates that range between 81,000 and 138,000. Snake venoms may cause a range of pathophysiological effects affecting the nervous system and the cardiovascular system. Moreover, snake venom may have tissue-damaging activities that result in lifelong morbidities such as amputations, muscle degeneration, and organ malfunctioning. The tissue-damaging components in snake venoms comprise multiple toxin classes with various molecular targets including cellular membranes and the extracellular matrix (ECM). In this study, we present multiple assay formats that enable investigation of snake venom-induced ECM degradation using a variety of (dye-quenched) fluorescently labeled ECM components. Using a combinatorial approach, we were able to characterise different proteolytic profiles for different medically relevant snake venoms, followed by identification of the responsible components within the snake venoms. This workflow could provide valuable insights into the key mechanisms by which proteolytic venom components exert their effects and could therefore prove useful for the development of effective snakebite treatments against this severe pathology.

## 1. Introduction

Snakebite envenoming is a neglected tropical disease that causes 81,000–138,000 fatalities annually worldwide [1]. The pathological effects of snakebite envenoming, resulting from the hemotoxic, neurotoxic, or cytotoxic properties of the venom, include tissue damage, paralysis, kidney failure, and hemorrhage [1,2,3,4]. These effects are generally caused by proteins and peptides, which are highly abundant in snake venom (>90% dry weight) [5]. The four major toxin classes are represented by snake venom phospholipase A2 (PLA_2_), three-finger toxins (3FTx), snake venom serine proteases (SVSPs), and snake venom metalloproteases (SVMPs). All other toxic components are classified as minor toxins [1,6]. PLA_2_s, SVMPs, 3FTxs, and some minor toxin classes cause tissue damage resulting in myonecrosis, damage of capillaries, rhabdomyolysis, and skin necrosis, which are evident in over 450,000 bite victims each year [1,4,7,8].

**Figure 1:**
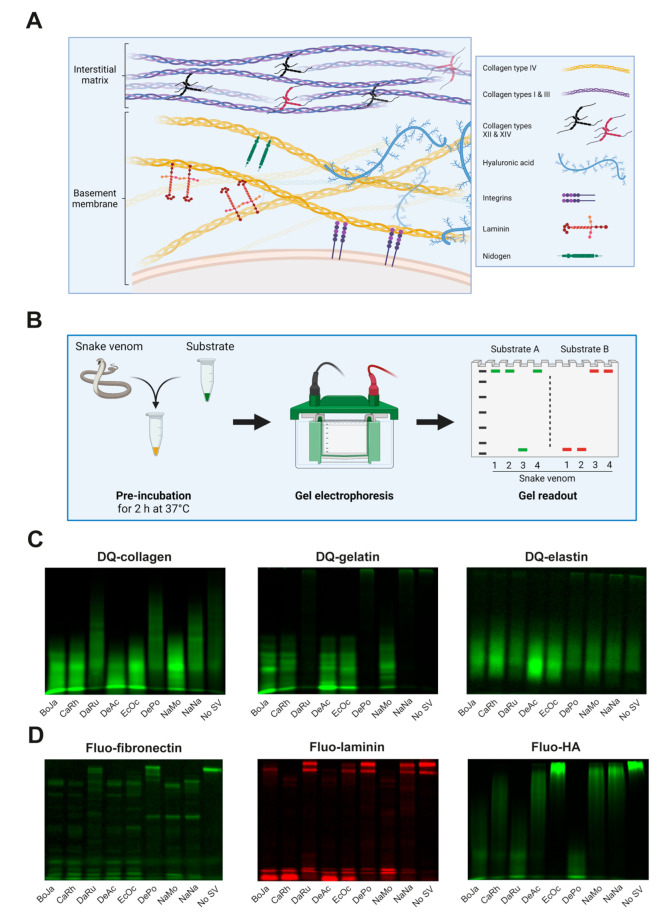
**Different ECM components and SDS-PAGE-based fluorescence detection of snake venom-induced degradation thereof.** (**A**) Schematic representation of the ECM composition. (**B**) Workflow used for the visualisation of fluorescent ECM substrate degradation patterns of different snake venoms. (**C**,**D**) SDS-PAGE-based visualisation of degradation of (**C**) dye-quenched fluorescent substrates DQ-collagen (40 µg/mL), DQ-gelatin (10 µg/mL), and DQ-elastin (100 µg/mL) or (**D**) non-dye-quenched (‘fluo-‘) fluorescent substrates fluo-fibronectin (40 µg/mL), fluo-laminin (40 µg/mL), and fluo-HA (50 µg/mL), after incubation with eight different indicated snake venoms (10 µg/mL for DQ-collagen, DQ-gelatin, fluo-fibronectin, and fluo-HA; 100 µg/mL for DQ-elastin and fluo-laminin) for 2 h at 37 °C. Abbreviations: BoJa, *B. jararaca*; CaRh, *C. rhodostoma*; DaRu, *D. russelii*; DeAc, *D. acutus*; EcOc, *E. ocellatus*; DePo, *D. polylepis*; NaMo, *N. mossambica*; NaNa, *N. naja*.

Tissue damage caused by snake venom toxins often involves degradation of substrates of the extracellular matrix (ECM) and surrounding tissues. The ECM plays a vital role in providing structural support to cells and acts as a filtration barrier, preventing infiltrating cells and larger molecules from passing through [9,10,11,12]. The ECM is made up of the interstitial matrix and the basement membrane. The former consists of various types of collagen (i.e., collagen types I, III, VI, XII, and XIV), whereas the latter consists of collagen types IV and VI, laminin, fibronectin, hyaluronic acid (HA), perlecan, and nidogen (Figure 1A) [9,10,11,12,13,14]. Due to its pivotal role in cell viability, the ECM provides a great target for toxins known to degrade key substrates of the ECM, such as SVMPs [10,14,15,16,17,18]. The most clinically relevant role of SVMPs is induction of local and systemic bleeding as a result of their proteolytic activity, destroying the ECM supporting the endothelial layer in capillary vessels [10,11,12,13,14]. 

Currently, only a limited number of studies have focused on the development of proteolytic assays that combine activity profiling with toxin identification [19,20,21,22]. In those cases that identification was performed via tryptic digestion followed by LC-MS/MS analysis, only a limited number of snake species was used [19,20]. In order to study the proteolytic activities of a selection of medically relevant snake venoms, here we used a variety of fluorescently labeled ECM substrates (gelatin, collagen, elastin, fibronectin, laminin, and hyaluronic acid). Using SDS-PAGE, we were able to study the activity patterns of crude venoms over time and identify the venom components responsible for substrate cleavage via the application of tryptic digestion followed by nanoLC-MS/MS. Using inhibitors of SVMPs validated the important role of these proteolytic toxins in ECM degradation. The data obtained in this study may prove particularly useful for the development of improved snakebite treatments, notably for those toxins that specifically target proteolytic components.

## 2. Material and Methods

### 2.1. Venoms

Venoms were sourced from the extensive library of the Faculty of Science, BioAnalytical Chemistry, Vrije Universiteit Amsterdam (VU). This library contains samples obtained and subsequently provided by the Liverpool School of Tropical Medicine (LSTM), National University of Singapore (NUS) and captive breeders. The snake venoms used in this study came from the following viper (Viperidae) and elapid (Elapidae) species: *Bothrops jararaca* (jararaca, captive bred); *Calloselasma rhodostoma* (Malayan pit viper, Thailand); *Daboia russelii* (Russell’s viper, locality unknown); *Deinagkistrodon acutus* (sharp-nosed viper, locality unknown); *Dendroaspis polylepis* (black mamba, captive bred); *Echis ocellatus* (West African carpet viper, Nigeria); *Naja mossambica* (Mozambique spitting cobra, captive bred); and *Naja naja* (Indian cobra, captive bred). These species were selected as they represent some of the most medically relevant species across the geographical regions most heavily affected by snakebite (i.e., Latin America, Sub-Saharan Africa and Southeast Asia). Venoms from VU and NUS were lyophilised immediately after milking, then freeze dried and stored at −80 °C. LSTM venoms were extracted, stored overnight at −20 °C, then lyophilised and stored at 4 °C long term. Samples were reconstituted in milliQ (mQ) H_2_O to the desired stock solutions, depending on the type of assay. These solutions were then aliquoted and subsequently snap frozen in liquid nitrogen and stored at −80 °C until use. All venoms were sourced in accordance with the Nagoya protocol, where applicable [23]. 

### 2.2. Chemicals and Reagents

Water was purified to Milli-Q water grade using an in-house Milli-Q^®^ Reference Water Purification System (Merck Millipore, Darmstadt, Germany). Gibco^TM^ Dulbecco’s phosphate-buffered saline (DPBS) (no calcium, no magnesium, pH 7.0–7.3) was purchased from Sigma-Aldrich (Zwijndrecht, The Netherlands). Various substrates were used, including DQ^TM^ gelatin from pig skin, fluorescein conjugate (Thermo Fisher Scientific, Ermelo, The Netherlands); DQ^TM^ collagen type I from bovine skin, fluorescein conjugate (Thermo Fisher Scientific); DQ^TM^ elastin from bovine neck ligament, BODIPY FL conjugate (Thermo Fisher Scientific); rhodamine laminin from Engelbreth-Holm–Swarm mouse tumor (Cytoskeleton, Inc., Denver, CO, USA); fibronectin from bovine plasma, HiLyte Fluor^TM^ 488 labeled (Cytoskeleton, Inc.); and fluorescein hyaluronic acid (Sigma-Aldrich). Positive controls included collagenase type IV from *Clostridium histolyticum* (Thermo Fisher Scientific); elastase from pig pancreas (Thermo Fisher Scientific); and hyaluronidase from bovine testes Type IV-S (Sigma-Aldrich). For the inhibitor assays, two small-molecule SVMP inhibitors were used, including marimastat (Sigma-Aldrich) and prinomastat (Sigma-Aldrich). 

### 2.3. Detection of Proteolytic Activity of Snake Venoms

For profiling of ECM substrate degradation patterns by different snake venoms, crude snake venoms were incubated with one of the fluorescent ECM substrates (substrate diluted in DPBS, concentration depending on the substrate, see figures) and incubated for 2 h at 37 °C. For kinetic measurements, DQ-gelatin (10 µg/mL) incubation with *C. rhodostoma* (10 µg/mL) was stopped at various time points. For DQ-gelatin inhibitor assays, *C. rhodostoma* (20 μg/mL, 2× pre-concentrated) was incubated with concentration–response curves (CRCs) of SVMP inhibitors marimastat or prinomastast for 30 min at 37 °C, before addition of DQ-gelatin (10 µg/mL final concentration) and incubation for another 2 h at 37 °C. For marimastat single-point inhibition assays with all fluorescent substrates, the most potent snake venom (10 or 100 μg/mL) was incubated with or without marimastat (1 µM) for 30 min at 37 °C. Subsequently, fluorescent substrates (final concentrations of 10 μg/mL for DQ-gelatin and DQ-collagen, 40 μg/mL for fluo-fibronectin and fluo-laminin, 50 μg/mL for fluo-HA and 100 μg/mL for DQ-elastin) were added to the mixtures, which were incubated for another 2 h at 37 °C. For all assays, incubation of snake venom with fluorescent substrate was stopped by addition of reducing Laemmli sample buffer (60 mM Tris-HCl, pH 6.8, 10% glycerol, 2% SDS, 0.01% bromophenol blue, 200 mM DTT) and subsequent heating of the samples for 5 min at 95 °C. After loading 20 μL of each sample on gel and performing gel electrophoresis, gels were washed in DPBS, and proteolytic activity of snake venoms was visualised using the Azure 400 fluorescent imager (Azure Biosystems, Dublin, USA). Data were captured at 472 nm excitation for all fluorescent substrates, except fluo-laminin for which 524 nm excitation was used. 

### 2.4. Identification of Proteolytically Active Proteins in Venoms Using In-Gel (Fluo-)Zymography

For in-gel zymography, 20 µg of crude snake venom was mixed with non-reducing Laemmli sample buffer (60 mM Tris-HCl, pH 6.8, 10% glycerol, 2% SDS, 0.01% bromophenol blue) and loaded on an SDS-PAGE gel, co-polymerised with gelatin or collagen. For conventional zymography, a 7.5% SDS-PAGE with gelatin (1 mg/mL) or collagen (1 mg/mL) was used, whereas for fluorescent zymography a 10% polyacrylamide gel including DQ-gelatin (63 µg/mL) or DQ-collagen (63 µg/mL) was used. After gel electrophoresis, gels were washed 3× for 30 min with Triton X-100 (2.5%) to remove SDS and (partially) renature the venom proteins for recovery of their enzymatic activity. Gels were subsequently washed 3× for 10 min with mQ-water to remove Triton X-100 prior to incubation. Conventional zymography gels were then incubated overnight at 37 °C in activity buffer (50 mM Tris-HCl, pH 7.5, 200 mM NaCl, 10 mM CaCl). After overnight (O/N) incubation, these conventional zymography gels were stained for 2 h using Coomassie Brilliant Blue solution and were de-stained using 40% ethanol/10% acetic acid for 4 × 30 min. Proteolytic bands appeared as a clear band on a dark background, whereas highly abundant protein bands appeared as bands darker than the background. Fluo-zymography gels were also incubated in activity buffer at 37 °C, but proteolytic activity was monitored in real time during O/N incubation. Imaging of all gels was performed using the Azure 400 fluorescent imager (Azure Biosystems, Dublin, CA, USA). For fluorescence imaging, 472 nm was used as excitation wavelength.

### 2.5. In-Gel Tryptic Digestion of Proteolytically Active Venom Components

To identify the bioactive components in the snake venoms, in-gel fluo-zymography was performed as described above, with the exception that 100 μg of snake venom was loaded instead of 20 µg, and protein bands of interest were carefully excised from the gels before being subjected to in-gel tryptic digestion. In order to make sure the correct bands were excised, a reference image of the gel was used, which was placed below the gel. Gel bands were washed consecutively with washing buffer (25 mM ammoniumbicarbonate pH = 8.2) and acetonitrile (ACN). After these washing steps, reduction buffer (0.05% β-mercaptoethanol in 25 mM ammonium bicarbonate, pH 8.2) was added, and the samples were incubated at 56 °C for 30 min. Alkylation buffer (55 mM iodoacetamide) was then added, and the gel bands were incubated for 20 min in the dark. Subsequently, a stock solution of trypsin (1 µg/µL in 50 mM acetic acid) was diluted 50 times in 25 mM ammonium bicarbonate to a concentration of 0.02 µg/mL and was added, after which the samples were incubated overnight at 37 °C. The following day, the samples were centrifuged at 1000 rpm for 1 min in an Eppendorf Centrifuge 5810 R (Hamburg, Germany) followed by adding 5% formic acid, in order to quench the reaction. 

### 2.6. Toxin Identification Using Proteomics

By subjecting the bioactive components to our proteomics workflow, we were able to identify the proteolytically active toxins in the venoms. The samples were analysed using nanoLC-MS/MS (or stored at −20 °C until analysis). Following the tryptic digestion, we performed nanoLC-MS/MS on the selected samples and used these samples for subsequent analysis. The samples were subjected to nano-LC separation on an UltiMate 3000 RSLCnano system (Thermo Fisher Scientific), followed by mass spectrometry. Mass spectrometry was performed on a maXis QTOF mass spectrometer (Bruker) carrying a Bruker Captive spray source that operates in positive-ion mode. Source parameters were source temperature: 150 °C; capillary voltage: 1.6 kV; dry gas flow: 3.0 L/min; and nanoBooster pressure: 0.20 Bar. The spectral data were recorded at 2 Hz (in a 50 to 3000 *m*/*z* range). MS/MS spectra were collected using collision-induced dissociation (CID) in data-dependent mode, which uses 10-eV collision energy. The data were processed using the Bruker DataAnalysis software, resulting in the identification of the proteins found in the individual venoms. 

Using the data for the tryptic digests of the samples, toxin identification was performed using MASCOT (Matrix Science, London, UK) searches against Swiss-Prot, NCBInr, and species-specific databases. The latter were generated from previously published transcriptomic data for all available species and were used for identification of those components for which proteolytic activity was observed. A more elaborate description of the entire approach can be found in the paper of Slagboom et al. (2020) [24]. For each sample (activity-associated band), we used a cutoff of 100 for the protein score and subsequently selected the three toxins with the highest protein scores. 

### 2.7. Statistical Analysis

Data were analysed using GraphPad Prism, Version 9.0.1 (GraphPad Software, San Diego, CA, USA). Figures were made with Adobe Illustrator 2020 (Adobe Systems, San Jose, CA, USA). The schematic figures illustrating assay formats were created with BioRender.com.

## 3. Results and Discussion

### 3.1. Profiling of Proteolytic Degradation Using SDS-PAGE Gels

We set out to develop an SDS-PAGE-based method for the detection and profiling of snake venom-induced degradation of different ECM substrates (Figure 1A). For this, we used a panel of medically relevant snake venoms in combination with a selection of fluorescently labeled, commercially available ECM substrates, aiming to maximise the accessibility of our newly developed method. Half of the fluorescent substrates, collagen type I, gelatin, and elastin, are dye-quenched (DQ) due to an excessive number of fluorophores and should therefore be essentially non-fluorescent in the absence of an active degrading enzyme. The other substrates, fibronectin, laminin, and hyaluronic acid (HA), are non-quenched (referred to as ‘fluo-…’) and display high levels of basal fluorescence. Upon pre-incubation with a substrate, the substrate-specific proteolytic activity in a snake venom was expected to yield (smaller) fluorescent degradation products that could be visualised using a fluorescent imager after performing SDS-PAGE (Figure 1B). 

The panel of eight snake venoms used throughout this work (Figure S1) displayed differential degradation patterns for the six substrates (Figure 1C,D). DQ-collagen type I and its derivative DQ-gelatin were entirely degraded by the crude venoms of *B. jararaca*, *C. rhodostoma*, *D. acutus*, *E. ocellatus*, and *N. mossambica*, as illustrated by the appearance of a low molecular weight smear or more well-defined degradation product bands, respectively (Figure 1C). *D. russelii*, *D. polylepis*, and *N. naja* were unable to degrade DQ-gelatin, whereas partial degradation of DQ-collagen by these venoms was apparent. Unexpectedly, for the third dye-quenched substrate, DQ-elastin, the presence of low molecular weight fluorescence in the condition without snake venom treatment (‘No SV’) was observed, indicating some basal degradation (Figure 1D). However, upon incubation with crude venoms from *B. jararaca*, *C. rhodostoma*, *D. acutus*, and *E. ocellatus*, pronounced enhancement of basal fluorescence could be detected. Fluo-fibronectin was degraded into a venom-specific combination of smaller fluorescent products by all venoms, although some residual intact substrate could be observed for *D. russelii* and *D. polylepis* (Figure 1D). Fluo-laminin was visible as two high molecular weight fluorescent bands in the absence of snake venoms (Figure 1D). Incubation with *B. jararaca*, *C. rhodostoma*, *D. acutus*, *E. ocellatus*, and *N. mossambica* resulted in (partial) proteolytic degradation of fluo-laminin, as evidenced by a reduction of the fluorescent starting material. The last substrate, fluo-HA, was most potently degraded by venom components of *D. polylepis*, to a lesser extent by those in *B. jararaca* and *D. russelii* venom, and only marginally by *C. rhodostoma*, *D. acutus*, *N. mossambica*, and *N. naja* venoms (Figure 1D). 

Next, we determined whether it was possible to monitor the degradation of two fluorescent ECM substrates with non-overlapping emission spectra simultaneously. For this, we used C. rhodostoma, which is amongst the species with the broadest proteolytic profile (Figure 1). In addition, we have used this species extensively in our previous studies; hence, arises our special interest in the activity patterns of and the active components in the venom of this particular species. We compared single incubations of *C. rhodostoma* with fluo-laminin or fluo-fibronectin with co-incubations of *C. rhodostoma* with one of the green substrates, DQ-gelatin, and with our only red substrate fluo-laminin, and measured fluorescence at 472 nm and 524 nm excitation (Figure S2). While the green fluo-fibronectin yielded a high amount of bleed-through in the red channel, this was not the case for DQ-gelatin. More importantly, due to size differences between intact DQ-gelatin and intact fluo-laminin as well as between degradation products of these two substrates, the co-incubation of DQ-gelatin and fluo-laminin with *C. rhodostoma* venom allowed for the simultaneous detection of their proteolytic degradation. Hence, our new SDS-PAGE-based method allows for differentiation between snake venoms based on their ECM substrate specificity and can be performed in a multiplexing fashion. 

### 3.2. Assessing Potency and Kinetics for Degradation of Fluorescent ECM Components

**Figure 2:**
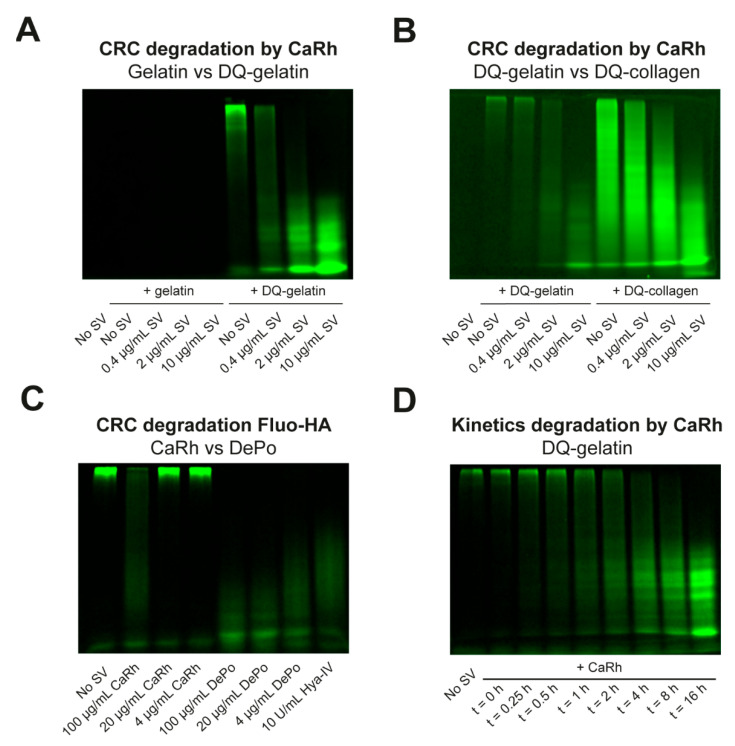
**Assessing potency and kinetics for degradation of fluorescent ECM components.** (**A**) SDS-PAGE-based visualisation of gelatin (10 µg/mL, left) or DQ-gelatin (10 µg/mL, right) degradation by concentration–response curve (CRC) of *C. rhodostoma* (CaRh) venom. (**B**) SDS-PAGE-based visualisation of DQ-gelatin (10 µg/mL, left) or DQ-collagen (10 µg/mL, right) degradation by CRC of *C. rhodostoma* venom. (**C**) SDS-PAGE-based visualisation of fluo-HA (50 µg/mL) degradation by CRC of *C. rhodostoma* (left) or *D. polylepis* venom (right), with Hya-IV (10 U/mL) as a positive control (most right column). (**D**) SDS-PAGE-based visualisation of kinetics for proteolytic degradation of DQ-gelatin (10 µg/mL) by *C. rhodostoma* (10 µg/mL).

Next, we assessed whether we could use the novel method to monitor potency and kinetics for proteolytic degradation of fluorescent ECM substrate by snake venoms. To test this, we used DQ-gelatin together with our model venom, *C. rhodostoma*. Increasing concentrations of *C. rhodostoma* venom yielded concentration-dependent degradation of DQ-gelatin but not unlabeled gelatin (Figure 2A). A similar pattern was observed for degradation of DQ-collagen by increasing concentrations of the same crude snake venom (Figure 2B). Next, we tested whether different potencies were detectable using this method. Therefore, we pre-incubated fluo-HA with two snake venoms: *C. rhodostoma* and *D. polylepis*. Complete degradation of fluo-HA by the positive control hyaluronidase-IV and all concentrations of *D. polylepis* venom was observed, whereas only incubation with the highest concentration of *C. rhodostoma* venom yielded partial degradation of fluo-HA (Figure 2C). Subsequently, we used different incubation times of *C. rhodostoma* venom with DQ-gelatin to determine the time course of DQ-gelatin degradation. The results indicated the proteolytic degradation of DQ-gelatin by *C. rhodostoma* was a time-dependent, saturable process (Figure 2D). Hence, potency and kinetics of fluorescent ECM substrate degradation by crude snake venoms can be assessed using the presented SDS-PAGE-based method. A clear limitation of the presented approach is the low throughput, and a much higher throughput for quantitative assessment of snake venom-induced degradation of ECM substrates could be achieved using plate reader-based assays that rely on a change in total fluorescence. However, this requires that the substrate is dye-quenched in the absence of an active degrading enzyme. For non-quenched substrates, proteolytic activity does not increase total fluorescence. The SDS-PAGE-based evaluation we report here, albeit having low throughput, allows for monitoring and quantification of degradation of these non-quenched substrates.

### 3.3. Extending the Method to Monitor Substrate-Specific Inhibition of ECM Degradation

ECM degradation by proteolytically active components in snake venoms is a key feature of the toxicity associated with snake bites. To prevent such toxicity, therapeutics that act as (non-) competitive enzyme inhibitors can be used. Given this, we assessed whether our new method, which combines SDS-PAGE with fluorescent ECM substrates, allows for monitoring specific inhibition of snake venom-induced ECM degradation. 

**Figure 3:**
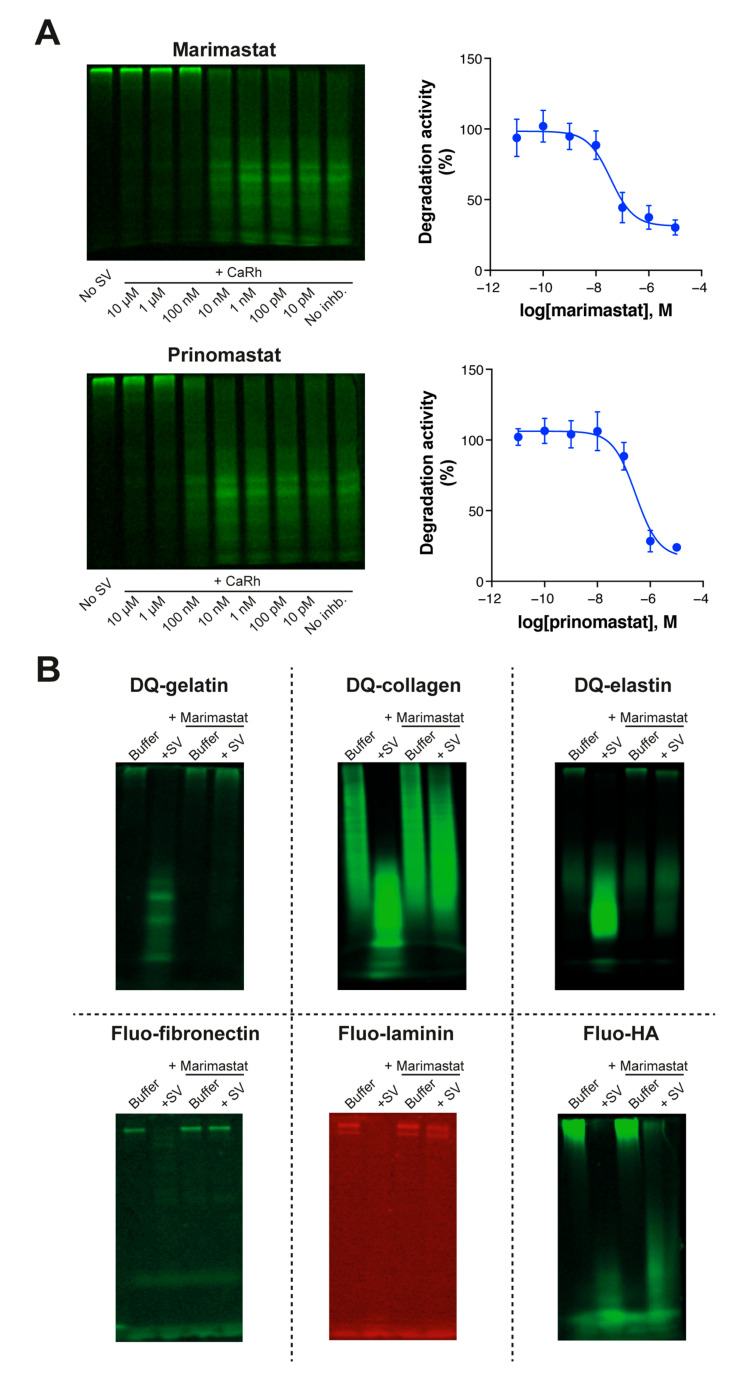
**Extending the method to monitoring substrate-specific inhibition of ECM degradation**. (**A**) SDS-PAGE-based visualisation and quantification of the inhibition of snake venom (*C. rhodostoma*)-induced proteolytic degradation of DQ-gelatin (10 μg/mL) by inhibitory CRCs of SVMP inhibitors marimastat and prinomastat. Inhibition was quantified by measuring the intensity of the degradation products and normalising these values to the ‘no SV’ condition (0%) and ‘SV no inhibitor’ condition (100%). Gels are representative of four individual experiments. Graphs depict the mean degradation activity ± SEM of these four individual experiments. (**B**) SDS-PAGE-based visualisation of snake venom-induced fluorescent substrate degradation inhibition by SVMP inhibitor marimastat. For each fluorescent ECM substrate, the most potent snake venom was pre-treated with or without marimastat (1 µM). For DQ-gelatin, DQ-collagen, and fluo-fibronectin this was *C. rhodostoma*; for DQ-Elastin and fluo-laminin this was *D. acutus*; and for fluo-HA this was *D. polylepis*.

Pre-treatment of *C. rhodostoma* venom with increasing concentrations of two SVMP inhibitors, marimastat and prinomastat, resulted in dose-dependent inhibition of DQ-gelatin proteolysis (Figure 3A). Marimastat (logIC_50_ = −7.4 ± 0.3) displayed an almost tenfold higher inhibitory potency compared to prinomastat (logIC_50_ = −6.6 ± 0.2). To evaluate the specificity of the observed inhibition, we then tested the inhibitory effects of marimastat on the snake venom-induced degradation of all six fluorescent ECM substrates. Although degradation of fluo-HA induced by the venom of *D. polylepis* remained largely unaffected by marimastat, snake venom-induced proteolytic degradation of all other substrates was found to be effectively inhibited by this inhibitor (Figure 3B). This suggests that SVMPs are primarily responsible for the observed degradation of all ECM substrates except HA. Hence, our method can be used to investigate the inhibitory potential and specificity of compounds targeting snake venom components. This could aid in identification of new compounds with therapeutic potential against specific toxins found in different snake venoms.

### 3.4. Identification of Proteolytic Components Using (Fluo-)Zymography

**Figure 4:**
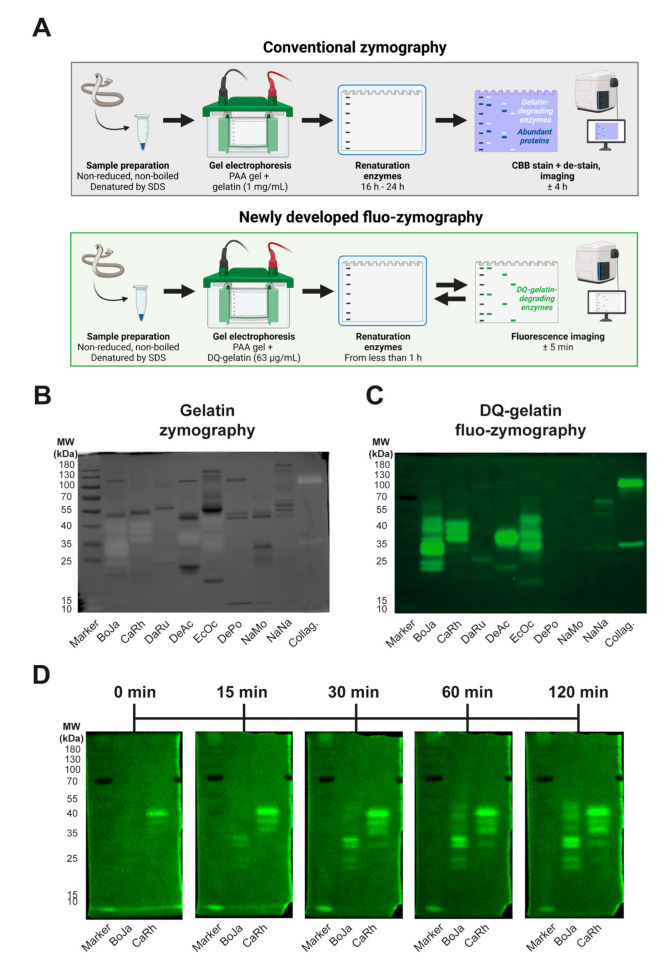
**Assessing the proteolytic activity of snake venoms using (fluo-)zymography.** (**A**) Schematic representation of the workflow of conventional zymography and the newly developed fluo-zymography methods. (**B**) Activity profiles for the conventional in-gel zymography (1 mg/mL of gelatin) of our panel of snake venoms (20 µg) after 16 h of incubation. (**C**) Activity profiles for the in-gel fluo-zymography (63 µg/mL of DQ-gelatin) of our panel of snake venoms (20 µg) after 2 h of incubation. (**D**) Kinetics of fluorescent substrate degradation for the two most potent snake venoms (i.e., *B. jararaca* and *C. rhodostoma*). Abbreviations: BoJa, *B. jararaca*; CaRh, *C. rhodostoma*; DaRu, *D. russelii*; DeAc, *D. acutus*; EcOc, *E. ocellatus*; DePo, *D. polylepis*; NaMo, *N. mossambica*; NaNa, *N. naja*.

In-gel zymography can be used to identify substrate-specific proteolytically active components within a mixture of proteins. Conventionally, non-fluorescent substrates, such as gelatin, are used for co-polymerisation with SDS-PAGE gels in order to obtain zymograms [21,22,25]. However, in an attempt to investigate whether it was possible to use dye-quenched fluorescent ECM substrates for in-gel-zymography, we named the process ‘fluo-zymography’. In theory, this approach would allow for real-time detection of in-gel proteolytic activity, which is not possible with conventional zymography (Figure 4A). For our panel of snake venoms, we performed in-gel conventional zymography and fluo-zymography using (DQ-)gelatin as the co-polymerised substrate (Figure 4B,C). In accordance with DQ-gelatin degradation profiles of the different snake venoms (Figure 1C), we observed the highest abundance and intensity of proteolytically active bands for all viper venoms except *D. russelii* (Figure 4B,C). Despite a more than 15-times-lower substrate concentration used for fluo-zymography and shorter incubation time (2 h vs. 16 h), a higher number of active bands were apparent using this novel method. This indicates a higher sensitivity of fluo-zymography than conventional zymography. Further comparison of conventional zymography and fluo-zymography gels revealed that the most abundant toxins (i.e., the bands that were most heavily stained with Coomassie Brilliant Blue) were not necessarily most active in terms of gelatin-degrading capacity (Figure 4B,C). While for (DQ-)collagen almost no activity bands could be monitored with conventional zymography using unlabeled collagen, fluo-zymography revealed a more pronounced degradation pattern when using DQ-collagen (Figure S3A,B). As expected, using two venoms with the highest DQ-gelatin degradation capacity (i.e., *B. jararaca* and *C. rhodostoma*) demonstrated that fluo-zymopgraphy allows for real-time monitoring of ECM substrate proteolytic degradation (Figure 4D). Interestingly, where for *C. rhodostoma* most proteolytically active bands were visible directly after renaturation, for *B. jararaca* the majority of the proteolytically active bands became apparent at later time points. In summary, the newly developed fluo-zymography approach (1) requires a lower ECM substrate concentration for co-polymerisation in SDS-PAGE gels, (2) seems to offer a higher sensitivity than conventional zymography (especially for collagen zymography), and (3) can capture the contributions of individual components within snake venoms over time, in contrast to conventional zymography.

### 3.5. Validation of Proteolytically Active Proteins within Crude Snake Venoms Using Proteomics

In general, viper venoms demonstrated more activity on the substrates compared to the elapid venoms, with the exception of *D. russelii* venom (Figure 4B,C). The (DQ-)gelatin-degrading profile of all proteolytically active vipers was characterised by low- to mid-weight compounds (i.e., 20–55 kDa), although some high mass compounds (i.e., >70–130 kDa) were also present, albeit displaying lower proteolytic activity (Figure 4C). The venom of *D. russelii* did not show a high level of substrate-degrading capacity, except for a few minor bands at varying molecular weights (i.e., around 100 kDa, 55 kDa, and 25 kDa). The elapid venoms showed considerably less activity, with *D. polylepis* venom being devoid of activity except for two minor bands around 40–55 kDa. The venoms of *N. mossambica* and *N. naja* showed somewhat higher activity, although the observed effect was lower when compared to the viper venoms. These findings were anticipated based on previous studies on proteolytic degradation by snake venoms [19,21,22,26]. 

**Figure 5:**
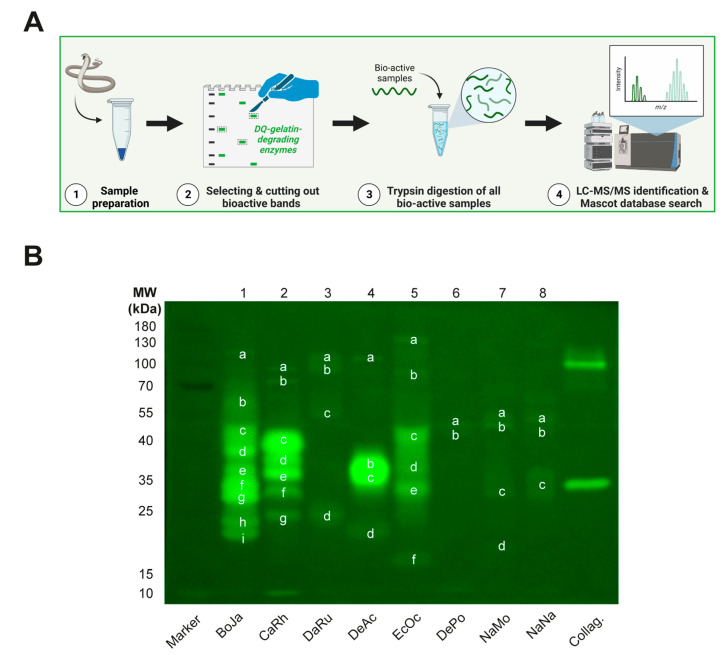
**Proteomics of ECM-degrading components in crude snake venoms using fluo-zymography.** (**A**) Schematic representation of the workflow for the identification of proteolytic components within crude snake venoms using proteomics. (**B**) Activity profiles for DQ-gelatin in-gel fluo-zymography of our panel of snake venoms (100 μg) and positive control collagenase (2 U) after 2 h of incubation. Letters depict bioactive bands in the venom of each species that were excised for subsequent toxin identification using proteomics. Abbreviations: BoJa, *B. jararaca*; CaRh, *C. rhodostoma*; DaRu, *D. russelii*; DeAc, *D. acutus*; EcOc, *E. ocellatus*; DePo, *D. polylepis*; NaMo, *N. mossambica*; NaNa, *N. naja*. The letters corresponding to each of the gel bands and the identified proteins can be found in Table S2.

For further analysis of those components responsible for the observed proteolytic activity, we combined fluo-zymography with advanced analytical techniques (Figure 5A). For this, we excised all bands containing bioactive proteins, followed by tryptic digestion of each sample and subsequent MS/MS analysis for toxin identification (Figure 5B). For each band, the three proteins with highest protein score were selected.

The proteomics results demonstrated that for the venoms with high DQ-gelatin-degrading capacity two toxin classes were more commonly represented, namely SVMPs and LAAOs. SVMPs form a class of enzymatic toxins that are found in all venomous snake families and which are subdivided into three groups (i.e., P-I to P-III) based on their domain structure [6,14,27]. The P-I group (~20–30 kDa) solely carries the metalloproteinase domain, whereas the P-II group (20–60 kDa) has an additional disintegrin domain, and the P-III group (60–100 kDa) has a disintegrin-like domain and an additional cysteine-rich domain [28,29]. In all viper venoms, we find SVMPs from all three subclasses. The substrate-degrading activity likely results from the SVMPs present in the venom, given the fact these toxins are highly abundant in the venoms (Table S1) and have the capacity of proteolytically degrading specific substrates [10,11,13]. This is in accordance with previous studies in which proteolytic activity of snake venoms was studied using zymogram gels [19,21,22,30,31,32,33]. Our inhibitor data (Section 3.3) further suggest that SVMPs are likely to be responsible for the observed degradation of most ECM substrates. Arguably, the most clinically relevant role of SVMPs is their induction of local and systemic bleeding by destruction of the ECM supporting the endothelial layer in capillary vessels [10,11,13]. SVMPs destabilise the interactions between endothelial cells and the basement membrane through degradation of key components in the endothelial cell membrane and the basement membrane [10,11,14,34]. The basement membrane degradation by SVMPs further affects a variety of other cell types, including skeletal muscle cells, keratinocytes, and kidney cells [1,10,35,36]. 

LAAOs is the second class of toxins that was regularly found in the viper venoms, and these are a group of enzymatic proteins made up of two subunits, each with a molecular weight of 50–70 kDa. The LAAOs are devoid of proteolytic activity and exert their cytotoxic effects by mechanisms unrelated to the degradation of key components. The clinical effects of LAAOs include haemolytic activity, hemorrhage, myotoxicity, and induction of oedema in various tissues [37,38,39,40]. Other toxin classes present in some of the viper venoms included snake venom serine proteases (SVSPs), C-type lectin-related proteins (i.e., a group of toxins that is also known by the name ‘snaclecs’), and PLA_2_s. These classes however have other activities and lack the ability to cleave ECM substrates [29,41,42,43,44,45,46]. 

Although the elapid venoms demonstrated much lower DQ-gelatin-degrading capacity on the fluo-zymograms compared to viper venoms, we did observe some proteolytic activity. For *N. mossambica* and *N. naja,* we found several P-III SVMPs, LAAOs, some cytotoxic 3FTxs, and two cysteine-rich proteins. For *D. polylepis* proteomics, we identified some SVMPs and a hyaluronidase; albeit, these Mascot hits belonged to a different genus (i.e *Bitis* and *Naja*) (Tables S2 and S3). 

## 4. Conclusions

This study describes the development of SDS-PAGE-based assay formats to visualise proteolytic degradation of fluorescent ECM substrates. These assays allowed characterisation of the proteolytic activity in venoms of a selection of medically relevant snake species. Viper venoms were generally more proteolytically active than elapid venoms, except for *D. russelii* venom. Although the venoms of *D. russelii* and *D. polylepis* were largely devoid of proteolytic activity, the HA-degrading activities in these species were noticeably and perhaps unexpectedly potent. The use of fluorescently labeled ECM substrates also facilitated investigation of the kinetics of proteolytic degradation by toxin components, while the use of small-molecule metalloproteinases inhibitors demonstrated that proteolysis could be readily inhibited in many cases, illustrating that the method could be implemented in efforts towards the identification of novel snakebite therapeutics. By developing and using fluo-zymography, we were able to identify the proteins within the crude snake venoms that were responsible for the observed proteolytic degradation. An advantage of our newly developed method of fluo-zymography over conventional zymography is the low substrate concentration required for co-polymerisation, which enhances the sensitivity of the approach and could be useful for ECM substrates that cannot be dissolved to high concentrations. In combination with proteomics on all active venom components, this approach provided us with an extensive overview of the cleavage patterns, dose-dependency, and kinetics of our panel of medically relevant species. Therefore, this workflow could be useful for studying toxins that specifically target the proteolytic components responsible for causing snakebite morbidity.

## Data Availability

Data is available upon request.

## Supplementary Materials

The following supporting information can be downloaded at: https://www.mdpi.com/article/10.3390/biology12060765/s1, Figure S1: Snake venom content overview; Figure S2: Multiplexing degradation of different ECM substrates; Figure S3: Conventional zymography vs fluo-zymography for collagen; Table S1: Overview of the 10 species included in the study with the proportion of the 10 major protein families in each venom (as percent of total venom); Table S2: Identification of toxins in gel band samples that show proteolytic activity for our panel of snake venoms after tryptic digestion after we used a cutoff of 100 for the protein score and subsequently selected the three toxins with the highest protein scores. Table S3: List of all toxins identified in gel band samples that show proteolytic activity for our panel of snake venoms after tryptic digestion.
