# Supplementary material for: Monitoring Snake Venom-Induced Extracellular Matrix Degradation and Identifying Proteolytically Active Venom Toxins Using Fluorescently Labeled Substrates
Source: Biology (Basel). 2023 May 24;12(6):765. doi: 10.3390/biology12060765 (PMC10295075; doi:10.3390/biology12060765)
Supplement: Supplementary file 1 [file biology-12-00765-s001.zip › Supplementary Materials/aa - Supplementary Materials_v2_incl track changes.docx]

**Supporting Information**

**
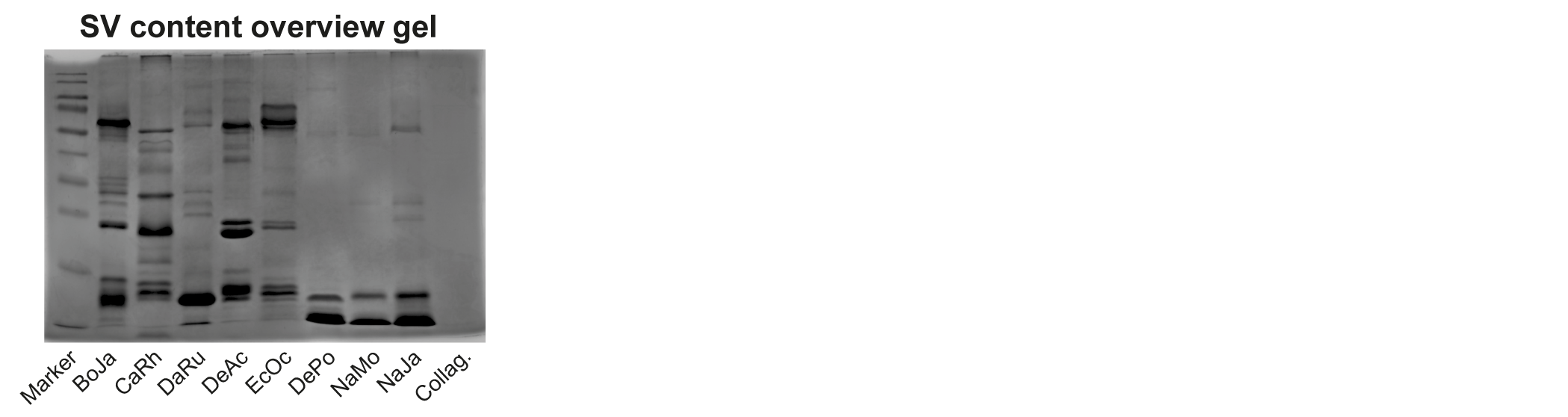
**

**Figure S1. Snake venom content overview.** Coomassie brilliant blue stain of SDS-PAGE gel (10%) containing reduced panel of snake venoms (20 µg).

**
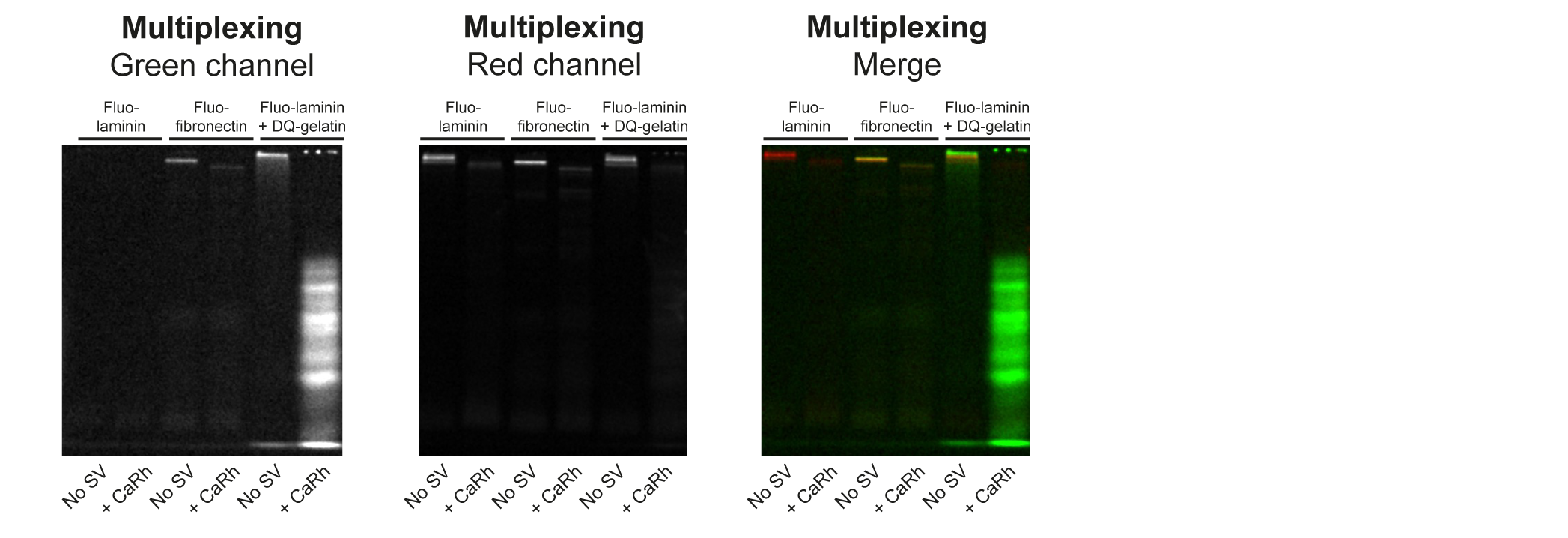
**

**Figure S2: Multiplexing degradation of different ECM substrates.** SDS-PAGE-based visualisation of degradation of single substrates fluo-laminin (50 µg/mL) and fluo-fibronectin (50 µg/mL) or dual degradation of fluo-laminin (50 µg/mL) and DQ-gelatin (10 µg/mL) by *C. rhodostoma* (100 µg/mL), co-incubated for 2 h at 37 °C. Fluorescence images from green channel (472 nm excitation), red channel (524 nm excitation) or merge of both channels.


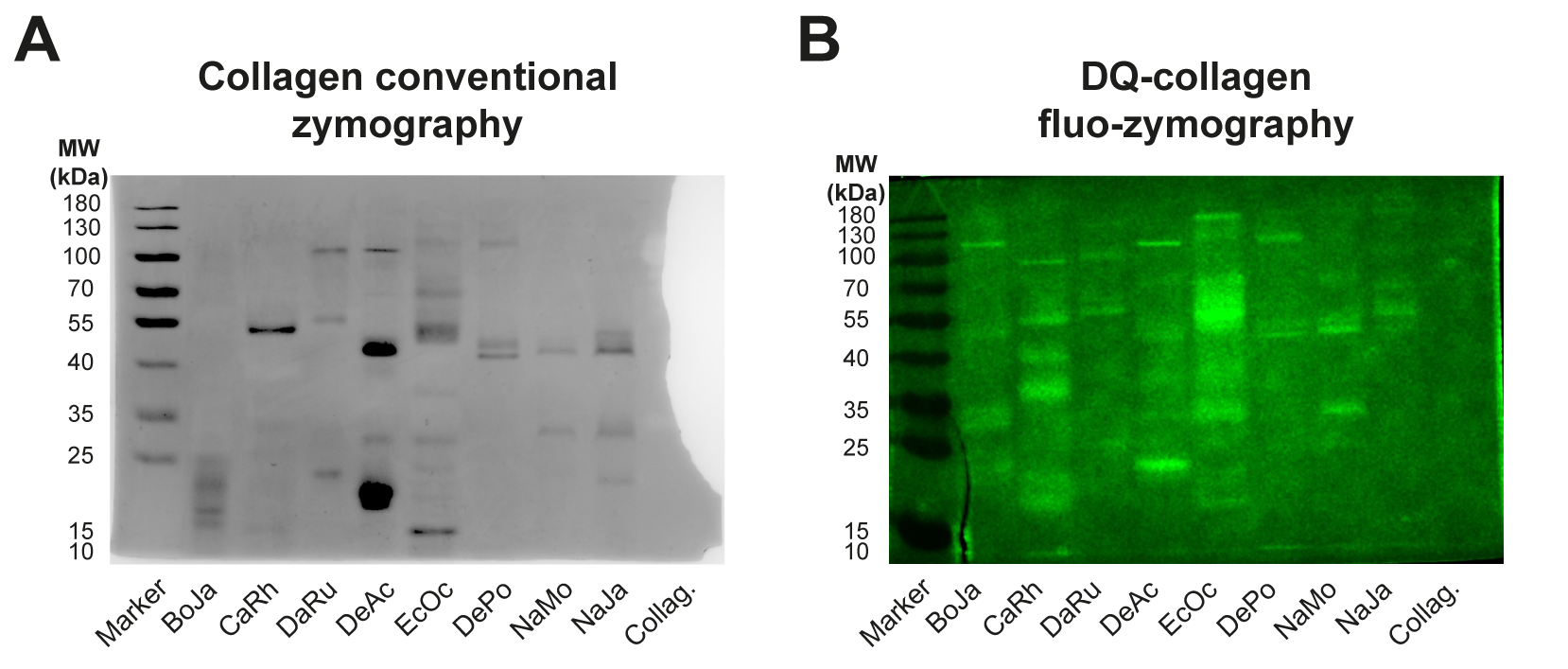


**Figure S3.** **Conventional zymography vs fluo-zymography for collagen. A)** Activity profiles for the conventional in-gel zymography (1 mg/mL of collagen type I) of our panel of snake venoms (20 µg) and positive control collagenase (2 U) after 16 h of incubation. **B)** Activity profiles for the in-gel fluo-zymography (63 µg/mL of DQ-collagen type I) of our panel of snake venoms (20 µg) after 2 h of incubation.

**Table S1. Overview of the 10 species included in the study with the proportion of the 10 major protein families in each venom (as percent of total venom).** Abbreviations: PLA_2_, phospholipase A2; SVSP, snake venom serine protease; SVMP, snake venom metalloprotease; LAAO, L-amino acid oxidase; 3FTx, three-finger toxin; KUN, Kunitz peptides; CRiSP, Cysteine-Rich Secretory Protein; NP, natriuretic peptide; %WV, percentage of venom.

| **Species** | **PLA_2_** | **SVSP** | **SVMP** | **LAAO** | **3FTx** | **KUN** | **CTL/SNACLEC** | **DIS** | **CRiSP** | **NP** | **%WV** | **Ref** |
| --- | --- | --- | --- | --- | --- | --- | --- | --- | --- | --- | --- | --- |
| ***B. jararaca*** | 3.7 - 20.2 | 13.7 - 28.6 | 10.3 - 35.6 | 7.2 - 8.0 |  |  | 9.4 - 9.6 | 0.2 - 7.0 | 2.4 - 2.6 | 16.4 - 22.6 | 95.6 - 100 | [1,2] |
| ***C. rhodostoma*** | 4.4 | 14.9 | 41.2 | 7 |  |  | 26.3 |  | 2.5 |  | 96.3 | [3] |
| ***D. russelii*** | 32.5 - 35 | 3.2 - 16 | 6.9 - 24.8 | 0.3 - 5.2 |  | 4.6 - 28.4 | 1.8 - 22.4 | 0 - 4.9 | 2 - 6.8 |  | 92.1 - 97.7 | [4–6] |
| ***D. acutus*** | 4.7 | 17.6 | 31.7 | 1.2 |  |  | 17.6 | 2.4 |  |  |  | [7] |
| ***E. ocellatus*** | 8.5 | 1.7 | 72.4 | 1.4 |  |  | 6.5 |  | 0.3 |  | 93.5 | [8] |
|  | | | | | | | | | | | | |
| ***D. polylepis*** |  |  | 3.2 |  | 31 | 61.1 |  |  |  | 2.9 |  | [9] |
| ***N. mossambica*** | 27.1 |  | 2.6 |  | 69.3 |  |  |  |  |  | 99 | [10] |
| ***N. naja*** | 11.4 - 21.4 | 0 - 0.3 | 0.9 - 1 | 0 - 0.8 | 63.8 - 80.5 | 0 - 0.4 |  |  | 2.1 - 3.7 | 0 - 2.0 | 79.8 - 99.1 | [11,12] |

Table S2. **Identification of toxins in gel band samples that show proteolytic activity for our panel of snake venoms after tryptic digestion after we used a cutoff of 100 for the protein score and subsequently selected the three toxins with the highest protein scores.** Table shows the species, the sample names, Mascot protein hits (i.e. UniProt Entry Names), protein scores, protein coverages (in %), toxin subclasses and peptide sequences. Abbreviations: SVMP, snake venom metalloprotease; LAAO, L-amino acid oxidase; SVSP, snake venom serine protease; PLA_2_, phospholipase A_2_; 3FTX, three-finger toxin.


**Table S3. List of all toxins identified in gel band samples that show proteolytic activity for our panel of snake venoms after tryptic digestion.** Table shows the species, protein accession (i.e. UniProt Entry Name), protein mass, protein coverage, protein description and peptide and protein sequence respectively.

References (for Table S1)

1. Gonçalves-Machado L, Pla D, Sanz L, Jorge RJB, Leitão-De-Araújo M, Alves MLM, et al. Combined venomics, venom gland transcriptomics, bioactivities, and antivenomics of two Bothrops jararaca populations from geographic isolated regions within the Brazilian Atlantic rainforest. J Proteomics. 2016;135: 73–89. doi:10.1016/J.JPROT.2015.04.029

2. Sousa LF, Nicolau CA, Peixoto PS, Bernardoni JL, Oliveira SS. Comparison of Phylogeny, Venom Composition and Neutralization by Antivenom in Diverse Species of Bothrops Complex. PLoS Negl Trop Dis. 2013;7: 2442. doi:10.1371/journal.pntd.0002442

3. Tang ELH, Tan CH, Fung SY, Tan NH. Venomics of Calloselasma rhodostoma, the Malayan pit viper: A complex toxin arsenal unraveled. J Proteomics. 2016;148: 44–56. doi:10.1016/j.jprot.2016.07.006

4. Kalita B, Patra A, Mukherjee AK. Unraveling the Proteome Composition and Immuno-profiling of Western India Russell’s Viper Venom for In-Depth Understanding of Its Pharmacological Properties, Clinical Manifestations, and Effective Antivenom Treatment. J Proteome Res. 2017;16: 583–598. doi:10.1021/acs.jproteome.6b00693

5. Tan NH, Fung SY, Tan KY, Yap MKK, Gnanathasan CA, Tan CH. Functional venomics of the Sri Lankan Russell’s viper (Daboia russelii) and its toxinological correlations. J Proteomics. 2015;128: 403–423. doi:10.1016/j.jprot.2015.08.017

6. Mukherjee AK, Kalita B, Mackessy SP. A proteomic analysis of Pakistan Daboia russelii russelii venom and assessment of potency of Indian polyvalent and monovalent antivenom. J Proteomics. 2016;144: 73–86. doi:10.1016/j.jprot.2016.06.001

7. Nie X, He Q, Zhou B, Huang D, Chen J, Chen Q, et al. Exploring the five-paced viper (Deinagkistrodon acutus) venom proteome by integrating a combinatorial peptide ligand library approach with shotgun LC-MS/MS. J Venom Anim Toxins Incl Trop Dis. 2021;27: 1–10. doi:10.1590/1678-9199-JVATITD-2020-0196

8. Casewell NR, Harrison RA, Wüster W, Wagstaff SC. Comparative venom gland transcriptome surveys of the saw-scaled vipers (Viperidae: Echis) reveal substantial intra-family gene diversity and novel venom transcripts. BMC Genomics. 2009;10: 1–12. doi:10.1186/1471-2164-10-564

9. Laustsen AH, Lomonte B, Lohse B, Fernández J, Gutiérrez JM. Unveiling the nature of black mamba (Dendroaspis polylepis) venom through venomics and antivenom immunoprofiling: Identification of key toxin targets for antivenom development. J Proteomics. 2015;119: 126–142. doi:10.1016/j.jprot.2015.02.002

10. Petras D, Sanz L, Segura Á, Herrera M, Villalta M, Solano D, et al. Snake venomics of African spitting cobras: Toxin composition and assessment of congeneric cross-reactivity of the Pan-African EchiTAb-Plus-ICP antivenom by antivenomics and neutralization approaches. J Proteome Res. 2011;10: 1266–1280. doi:10.1021/pr101040f

11. Dutta S, Chanda A, Kalita B, Islam T, Patra A, Mukherjee AK. Proteomic analysis to unravel the complex venom proteome of eastern India Naja naja: Correlation of venom composition with its biochemical and pharmacological properties. J Proteomics. 2017;156: 29–39. doi:10.1016/J.JPROT.2016.12.018

12. Sintiprungrat K, Watcharatanyatip K, Senevirathne WDST, Chaisuriya P, Chokchaichamnankit D, Srisomsap C, et al. A comparative study of venomics of Naja naja from India and Sri Lanka, clinical manifestations and antivenomics of an Indian polyspecific antivenom. J Proteomics. 2016;132: 131–143. doi:10.1016/J.JPROT.2015.10.007
