# Supplementary material for: Monitoring Snake Venom-Induced Extracellular Matrix Degradation and Identifying Proteolytically Active Venom Toxins Using Fluorescently Labeled Substrates
Source: Biology (Basel). 2023 May 24;12(6):765. doi: 10.3390/biology12060765 (PMC10295075; doi:10.3390/biology12060765)
Supplement: Supplementary file 1 [file biology-12-00765-s001.zip › Supplementary Materials/Supplementary Tables/S1 Table.docx]

| **SPECIES** | **PLA_2_** | **SVSP** | **SVMP** | **LAAO** | **3FTx** | **KUN** | **CTL/SNACLEC** | **DIS** | **CRiSP** | **NP** | **%WV** | **Ref** |
| --- | --- | --- | --- | --- | --- | --- | --- | --- | --- | --- | --- | --- |
| ***B. jararaca*** | 3.7 - 20.2 | 13.7 - 28.6 | 10.3 - 35.6 | 7.2 - 8.0 |  |  | 9.4 - 9.6 | 0.2 - 7.0 | 2.4 - 2.6 | 16.4 - 22.6 | 95.6 - 100 | [1,2] |
| ***C. rhodostoma*** | 4.4 | 14.9 | 41.2 | 7 |  |  | 26.3 |  | 2.5 |  | 96.3 | [3] |
| ***D. russelii*** | 32.5 - 35 | 3.2 - 16 | 6.9 - 24.8 | 0.3 - 5.2 |  | 4.6 - 28.4 | 1.8 - 22.4 | 0 - 4.9 | 2 - 6.8 |  | 92.1 - 97.7 | [4–6] |
| ***D. acutus*** | 4.7 | 17.6 | 31.7 | 1.2 |  |  | 17.6 | 2.4 |  |  |  | [7] |
| ***E. ocellatus*** | 8.5 | 1.7 | 72.4 | 1.4 |  |  | 6.5 |  | 0.3 |  | 93.5 | [8] |
|  |  |  |  |  |  |  |  |  |  |  |  |  |
| ***D. polylepis*** |  |  | 3.2 |  | 31 | 61.1 |  |  |  | 2.9 |  | [9] |
| ***N. mossambica*** | 27.1 |  | 2.6 |  | 69.3 |  |  |  |  |  | 99 | [10] |
| ***N. naja*** | 11.4 - 21.4 | 0 - 0.3 | 0.9 - 1 | 0 - 0.8 | 63.8 - 80.5 | 0 - 0.4 |  |  | 2.1 - 3.7 | 0 - 2.0 | 79.8 - 99.1 | [11,12] |

1. Gonçalves-Machado L, Pla D, Sanz L, Jorge RJB, Leitão-De-Araújo M, Alves MLM, et al. Combined venomics, venom gland transcriptomics, bioactivities, and antivenomics of two Bothrops jararaca populations from geographic isolated regions within the Brazilian Atlantic rainforest. J Proteomics. 2016;135: 73–89. doi:10.1016/J.JPROT.2015.04.029

2. Sousa LF, Nicolau CA, Peixoto PS, Bernardoni JL, Oliveira SS. Comparison of Phylogeny, Venom Composition and Neutralization by Antivenom in Diverse Species of Bothrops Complex. PLoS Negl Trop Dis. 2013;7: 2442. doi:10.1371/journal.pntd.0002442

3. Tang ELH, Tan CH, Fung SY, Tan NH. Venomics of Calloselasma rhodostoma, the Malayan pit viper: A complex toxin arsenal unraveled. J Proteomics. 2016;148: 44–56. doi:10.1016/j.jprot.2016.07.006

4. Kalita B, Patra A, Mukherjee AK. Unraveling the Proteome Composition and Immuno-profiling of Western India Russell’s Viper Venom for In-Depth Understanding of Its Pharmacological Properties, Clinical Manifestations, and Effective Antivenom Treatment. J Proteome Res. 2017;16: 583–598. doi:10.1021/acs.jproteome.6b00693

5. Tan NH, Fung SY, Tan KY, Yap MKK, Gnanathasan CA, Tan CH. Functional venomics of the Sri Lankan Russell’s viper (Daboia russelii) and its toxinological correlations. J Proteomics. 2015;128: 403–423. doi:10.1016/j.jprot.2015.08.017

6. Mukherjee AK, Kalita B, Mackessy SP. A proteomic analysis of Pakistan Daboia russelii russelii venom and assessment of potency of Indian polyvalent and monovalent antivenom. J Proteomics. 2016;144: 73–86. doi:10.1016/J.JPROT.2016.06.001

7. Nie X, He Q, Zhou B, Huang D, Chen J, Chen Q, et al. Exploring the five-paced viper (Deinagkistrodon acutus) venom proteome by integrating a combinatorial peptide ligand library approach with shotgun LC-MS/MS. J Venom Anim Toxins Incl Trop Dis. 2021;27: 1–10. doi:10.1590/1678-9199-JVATITD-2020-0196

8. Casewell NR, Harrison RA, Wüster W, Wagstaff SC. Comparative venom gland transcriptome surveys of the saw-scaled vipers (Viperidae: Echis) reveal substantial intra-family gene diversity and novel venom transcripts. BMC Genomics. 2009;10: 1–12. doi:10.1186/1471-2164-10-564

9. Laustsen AH, Lomonte B, Lohse B, Fernández J, Gutiérrez JM. Unveiling the nature of black mamba (Dendroaspis polylepis) venom through venomics and antivenom immunoprofiling: Identification of key toxin targets for antivenom development. J Proteomics. 2015;119: 126–142. doi:10.1016/j.jprot.2015.02.002

10. Petras D, Sanz L, Segura Á, Herrera M, Villalta M, Solano D, et al. Snake venomics of African spitting cobras: Toxin composition and assessment of congeneric cross-reactivity of the Pan-African EchiTAb-Plus-ICP antivenom by antivenomics and neutralization approaches. J Proteome Res. 2011;10: 1266–1280. doi:10.1021/pr101040f

11. Dutta S, Chanda A, Kalita B, Islam T, Patra A, Mukherjee AK. Proteomic analysis to unravel the complex venom proteome of eastern India Naja naja: Correlation of venom composition with its biochemical and pharmacological properties. J Proteomics. 2017;156: 29–39. doi:10.1016/J.JPROT.2016.12.018

12. Sintiprungrat K, Watcharatanyatip K, Senevirathne WDST, Chaisuriya P, Chokchaichamnankit D, Srisomsap C, et al. A comparative study of venomics of Naja naja from India and Sri Lanka, clinical manifestations and antivenomics of an Indian polyspecific antivenom. J Proteomics. 2016;132: 131–143. doi:10.1016/J.JPROT.2015.10.007
