# Supplementary figures and images for: Monitoring Snake Venom-Induced Extracellular Matrix Degradation and Identifying Proteolytically Active Venom Toxins Using Fluorescently Labeled Substrates
Source: Biology (Basel). 2023 May 24;12(6):765. doi: 10.3390/biology12060765 (PMC10295075; doi:10.3390/biology12060765)

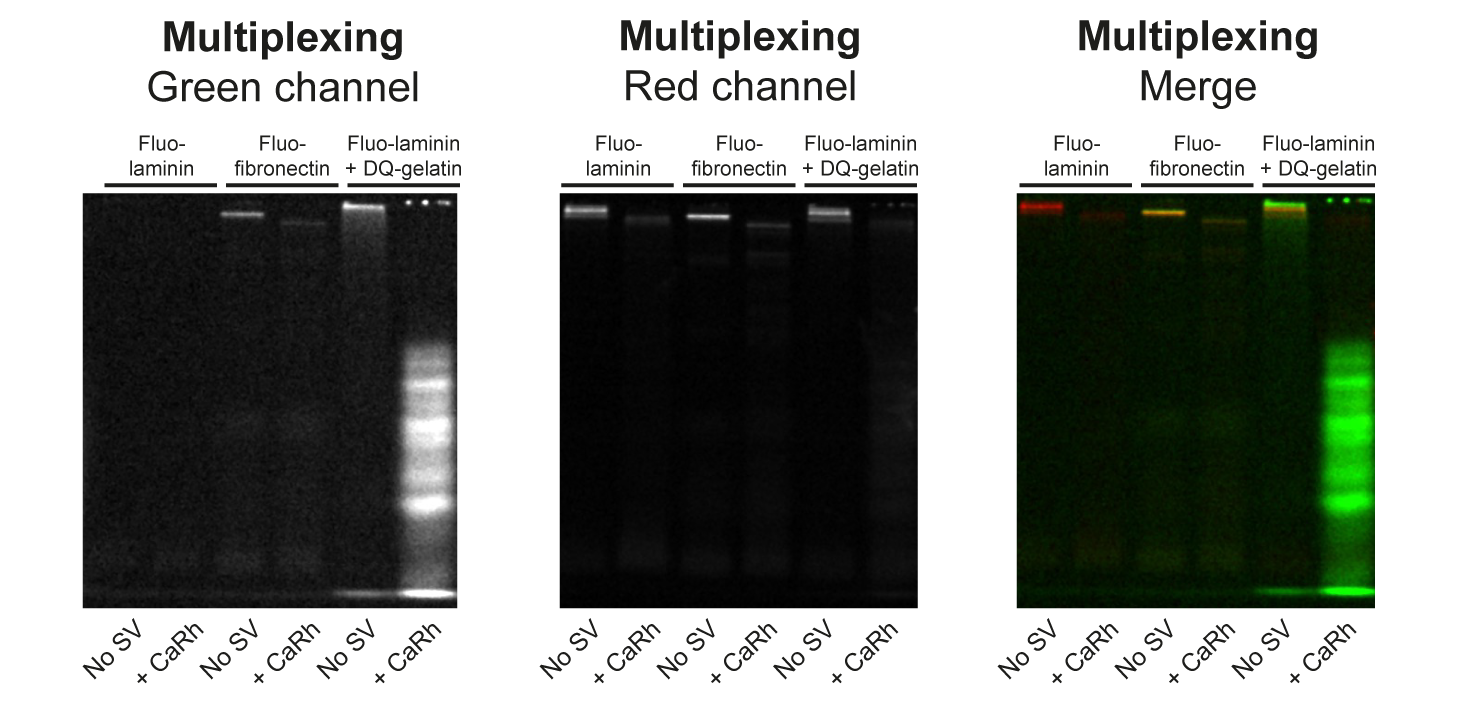

Supplement: Supplementary file 1 [file biology-12-00765-s001.zip › Supplementary Materials/Supplementary figures/Figures Matyas and Nick SV manuscript 1 v10 (PROOFS)_Figure S2.tif]

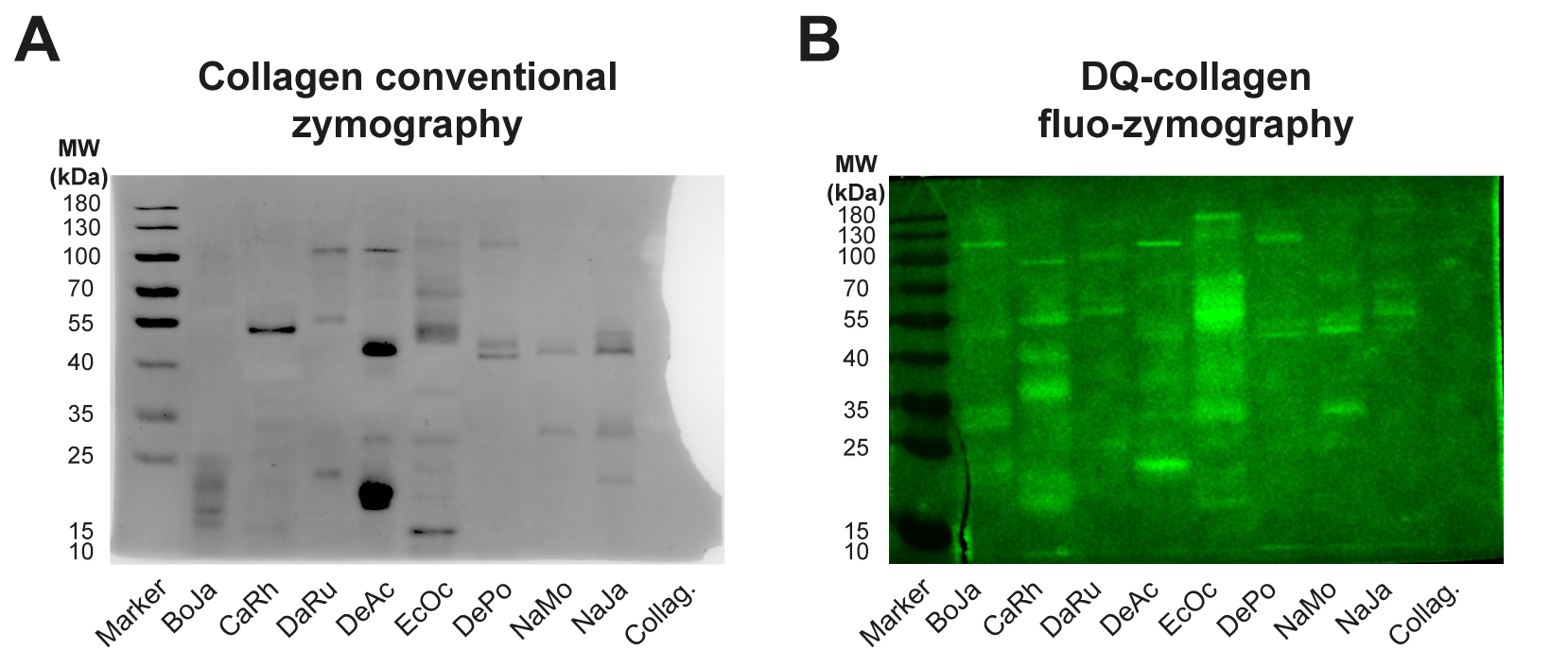

Supplement: Supplementary file 1 [file biology-12-00765-s001.zip › Supplementary Materials/Supplementary figures/Figures Matyas and Nick SV manuscript 1 v10 (PROOFS)_Figure S3.tif]

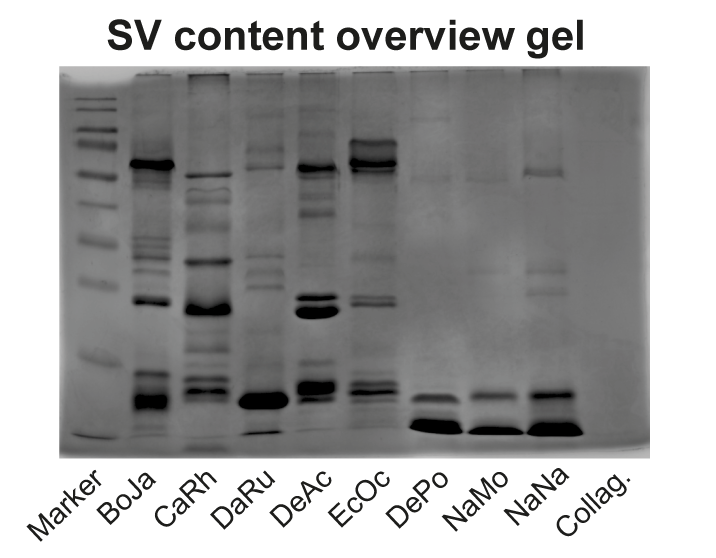

Supplement: Supplementary file 1 [file biology-12-00765-s001.zip › Supplementary Materials/Supplementary figures/Figures Matyas and Nick SV manuscript 1 v10 (PROOFS)_Figure S1.tif]
